# Increase in the Inflammatory Marker GlycA over 13 Years in Young Adults Is Associated with Poorer Cognitive Function in Midlife

**DOI:** 10.1371/journal.pone.0138036

**Published:** 2015-09-25

**Authors:** Irit Cohen-Manheim, Glen M. Doniger, Ronit Sinnreich, Ely S. Simon, Ronit Pinchas-Mizrachi, James D. Otvos, Jeremy D. Kark

**Affiliations:** 1 Hebrew University-Hadassah Braun School of Public Health and Community Medicine, Ein Kerem, Jerusalem, 91120, Israel; 2 Department of Clinical Research, NeuroTrax Corporation, Modiin, 71711, Israel; 3 Center for Medical Decision Making, Ono Academic College, Kiryat Ono, 55000, Israel; 4 Department of Neurology, Albert Einstein College of Medicine, the Bronx, New York City, New York, 10461, United States of America; 5 LabCorp, Raleigh, North Carolina, 27616, United States of America; Kessler Foundation Research Center, UNITED STATES

## Abstract

**Background:**

Inflammatory markers are elevated in patients with dementia. Evidence for an association between inflammation and cognitive function in dementia-free individuals is sparse, inconsistent, and predominantly restricted to the elderly. Assessment of inflammatory markers in young adults as predictors of cognitive function in midlife, well before the onset of overt dementia, is lacking. Furthermore, rarely has the relation with longitudinal change in inflammatory markers been examined.

**Objective:**

To examine the association of the inflammatory markers C-reactive protein (CRP), fibrinogen, white blood cell count (WBC) and GlycA, a novel NMR-determined biomarker of systemic inflammation, measured in young adulthood and of GlycA change over 13 years follow-up with cognitive function in midlife.

**Methods:**

507 participants of the Jerusalem Lipid Research Clinic (LRC) study were assessed at 3 time points over 18–22 years. First, the inflammatory variables GlycA, CRP, fibrinogen, and WBC were measured in blood samples drawn at ages 28–32. Then, in blood samples drawn a mean 13 years later (range, 12–16 years) at ages 41–46, GlycA was again measured (in 484 individuals). Subsequently at ages 48–52, on average 7 years later, global cognitive function and its five specific component domains were assessed with a NeuroTrax computerized test battery. Multiple regression and multivariable logistic models were applied.

**Results:**

Inverse unadjusted associations were shown for baseline levels and longitudinal change in inflammatory markers and measures of cognition. Multiple regression models were adjusted for age at cognitive assessment, sex, socio-demographic characteristics, baseline measures of leisure-time vigorous activity, smoking status and body mass index (BMI) at ages 28–32, change in smoking status and BMI between ages 28–32 and 41–46, and depression assessed at the time of cognitive testing. The highest quintile of GlycA change, but not the baseline inflammation measures, was inversely related to global cognition (standardized β = -.109, p = .011) as well as to the information processing speed and memory domains (standardized β = -.124, p = .008 and-.117, p = .014, respectively). The multivariable-adjusted odds ratio for low ranked global cognitive function (lowest fifth) comparing the extreme quintiles of GlycA change was 4.8 (95%CI, 1.7–13.5, p = .003; p for trend = .031).

**Conclusions:**

In this longitudinal study of a novel systemic inflammatory marker in a population-based cohort of young adults, GlycA increase over 13 years, but not baseline measures of inflammation, was associated with poorer cognitive function in midlife.

## Introduction

Inflammatory markers, including acute-phase inflammatory reactants, have been found in the cerebrospinal fluid and β-amyloid plaques in patients with dementia [1]. There is also evidence that high circulating levels of inflammatory markers are associated with the risk of incident dementia [2–4].

Evidence of an association between inflammatory markers and cognitive function in non-demented individuals is inconsistent and predominantly based on older adults, where preclinical dementia cannot be ruled out. These studies, which largely examined C-reactive protein (CRP) and IL-6, either showed no association [5–7], poorer function [8–15] or greater cognitive decline [16–19] in those with higher measures of inflammation. Some report associations with CRP but not IL-6 [8, 13], others with IL-6 but not CRP [16, 17, 19], and some find both to be associated with cognition [9, 11, 12, 14]. It remains unclear whether there are more relevant markers of peripheral inflammation in relation to cognitive function. Several studies reported associations with the white blood cell count (WBC) [10] and fibrinogen [18, 20]. Fewer still examined longitudinal change in inflammatory markers in relation to cognition [14, 17]. Studies of inflammatory markers measured in young adults as predictors of cognitive function in midlife, well before the onset of overt dementia, are lacking. There are no reports on the association of GlycA, a novel NMR-determined composite biomarker of systemic inflammation [21], or of GlycA change, with cognition.

The GlycA NMR signal originates from methyl group protons of a subset of glycan N-acetylglucosamine residues on enzymatically glycosylated acute phase proteins. As the measured amplitudes of this signal reflect the extent of plasma protein glycosylation, GlycA levels can serve as a nonspecific marker of global inflammation status [21].

In the present study, we examine a key question of predictors of cognitive function in young to middle-aged adults using 4 markers of systemic inflammation, CRP, fibrinogen, WBC and GlycA, the latter measured twice in young adulthood over a 13-year period. As long-term factors such as obesity [22], smoking [23] and socioeconomic status (SES) may play a role in the modulation of the immune system [24], we accounted in the analyses for body mass index (BMI), smoking status, childhood SES and adult SES as well as their changes over time.

Our objective was to examine, in a population-based cohort, the association of the inflammatory markers CRP, fibrinogen, WBC and GlycA, measured at baseline at ages 28–32, and of change in GlycA, measured on average 13 years later (range, 12–16 years) at ages 41–46 years, with global cognitive function, the primary outcome variable, determined at ages 48–52. The specific cognitive domains that contribute to global function served as secondary outcomes.

## Materials and Methods

### Study sample

In 1976–1978 (phase 1) the Jerusalem Lipid Research Clinic (LRC) Study initially examined 8646 17-y-old Jewish residents of Jerusalem, representing full age cohorts. A subsample comprising 1892 youngsters was reexamined within 3 months (phase 2). Details regarding sampling and response rates are available [25]. In 1980–1983 (phase 3), 4997 (58%) of the individuals screened at visit 1 were reexamined at age 20–22 y. In 1989–1991 (phase 4, the baseline of the current study), a sample numbering 1052 eligible subjects (686 men, 366 women) was examined and interviewed at age 28–32 y [26]. In 2003–2006 (phase 5, the follow-up of the present study), 631 of the baseline subjects were reexamined [27] [71% response rate, after exclusion of 168 ineligible participants who were not current Jerusalem residents [n = 154 (92 moved away from the city and 62 were abroad)], were pregnant or were within 3 mo of delivery (n = 4), had a serious incapacitating illness (e.g., metastatic cancer, end-stage renal disease, n = 3) or had died (n = 7)]. The inflammatory markers CRP, WBC and fibrinogen were measured at baseline and GlycA was assayed both in baseline and follow-up samples. In 2009–2011 (phase 6), 507 of the 631 participants underwent assessment of cognitive function (82% response rate, after exclusion of 13 ineligible participants who were not current Jerusalem residents, had a serious illness or had died).

The Jerusalem LRC study was approved by the Hadassah Medical Center Helsinki Ethics Committee. Participants provided signed informed consent.

### Cognitive function

Cognitive functions were assessed through a battery of NeuroTrax computerized cognitive tests (previously known as “MindStreams”) (NeuroTrax Corp., Modiin, Israel). The battery was designed to evaluate performance across an array of cognitive domains known to deteriorate during aging (including memory, executive function, visual spatial processing, attention, and information processing speed) and provide measurements of accuracy and response time in milliseconds shown to be valid [28, 29] and reliable [30] in a short administration time of approximately 30 minutes (0:32±0:04 in our study). Guidance and feedback were provided in practice sessions that preceded each test. All responses were made using the mouse buttons or with the number pad on the keyboard. Several of these tests are based on common neuropsychological paradigms (including the Benton Visual Retention Test, Brief Visuospatial Memory Test, Tova, Stroop, and subsets of WAIS-III (Wechsler Adult Intelligence Scale, 3rd ed.) and have been previously used in clinical settings, as well as in studies of normal aging relating these neuropsychological measurements to genetic findings [31] and brain imaging parameters [32], and have been used in middle-aged adults [31–35]. A detailed description of the tests can be found in S1 File.

### Inflammatory factors

Baseline and follow-up venous blood samples drawn after a 12-h overnight fast were stored at -80°C until analysis. Inflammation markers assessed at baseline were plasma concentrations of CRP (by ELISA) and fibrinogen (Clauss method), and WBC (Beckman Coulter Counter). Plasma GlycA (by proton nuclear magnetic resonance spectroscopy) was quantified in both baseline and follow-up samples, using an identical GlycA spectral deconvolution algorithm, as previously described [21].

### Covariates

Socio-demographic characteristics consisted of age, sex, origin (father's country of birth grouped into Europe, Asia, North Africa, and Israel), religiosity (ultra-orthodox, religious, traditional, secular), highest educational attainment (university degree, high school graduate, incomplete high school, elementary), childhood SES (2 measures based on father's occupation modified from the Israel Central Bureau of Statistics (ICBS), ranked similarly to the British Registrar General's scale [25], and the Vered Kraus Scale [36]) and adult SES (2 measures based on the modified ICBS ranking and the MacArthur Scale of Subjective Social Status [37]). Social mobility was computed by subtracting the ICBS-based SES in adulthood from the childhood SES measure (both having a range from 1 (upper) to 6 (lower)). The social mobility score, ranging from -5 (maximal downward drifting) to +5 (maximal upward mobility), was grouped as no change/upward mobility (scores ≥0) and downward drifting (scores <0).

Measures of BMI (in kg/m^2^), blood pressure, heath behaviors and biochemistry were obtained from the baseline examination and interview at mean age 30y. Blood pressure was taken as the mean of the last 2 of 3 seated measurements using a standard mercury sphygmomanometer after 5 min of quiet rest. Health behaviors consisted of cigarette smoking status (never, former, current), alcohol intake (≤ once a week, and a median split of number of units per week as 2 dummy variables of low and high intake), and vigorous physical leisure-time activity for at least 20 minutes causing sweating and shortness of breath (yes/no). Smoking status and BMI were also obtained from the follow-up visit at mean age 43y. Smoking status change was classified to no change, change from former to current smoker or current to former smoker as dummy variables. BMI change was defined as the mean difference between measurements. Plasma glucose, insulin, total cholesterol, HDL-C and triglycerides were measured on samples drawn after a 12-h fast by standard enzymatic techniques and radioimmunoassay (insulin). LDL-C was computed by the Friedewald method [38]. Serum homocysteine level was determined using HPLC with fluorometric detection.

Depressive and anxiety symptoms were measured at phase 6 using a translated Hebrew version of the Hospital Anxiety and Depression Scale (HADS) (two 7-item independent subscales)[39].

### Statistical analysis

Cognitive raw outcome measures (i.e., response time, accuracy and composite scores) were z-standardized to permit the averaging of performances across the different types and scales of the outcome measures. Timed measures (response time and response time SD) were multiplied by -1 so that higher values indicate better performance. The z-standardized measures were then averaged to produce five scores, each indexing a different cognitive domain: memory, attention, executive function, visual spatial, and information processing speed. A summary global cognitive score, computed as the average of the 5 domain scores, was treated as the main dependent variable. Cognitive scores with negatively skewed distributions (global, memory and attention) were Box-Cox [40] power transformed (λ = - 0.5), i.e., inverse square-root transformed, to achieve an approximately Gaussian distribution, subsequent to reflection (computed by subtracting each value of a negatively skewed score from a constant).

WBC and CRP which showed positive skewness underwent a Box-Cox transformation (λ = 0) that did not materially affect the associations of WBC (and is not reported) and only the transformed CRP is reported. Baseline and follow-up GlycA, GlycA change and fibrinogen, were approximately normally distributed.

Multiple linear regression models were used to examine the associations between each inflammatory marker (independent variable) and global function (the dependent variable). These models were repeated separately for each of the 5 cognitive domains to assess to which component(s) the association with global function can be attributed. Regression coefficients are reported as standardized betas (β). Odds ratios (ORs) and 95% confidence intervals (CI) for poor cognitive function associated with inflammation were computed from logistic models. A score in the lowest quintile was classified as relatively poor cognitive performance. Longitudinal GlycA change was modeled as a predictor variable in three modes: as a continuous variable, as quintiles of change, and as a dichotomous variable (the highest quintile of change vs the lowest 4 grouped). Tests for linear trend across quintiles were performed by including an ordinal variable with the median value of each quintile introduced in the regression models.

Analyses were adjusted for age, sex, education, origin, SES in childhood (ICBS ranking), adult SSES (ICBS ranking), baseline leisure-time vigorous activity, smoking status, BMI, and depression. These were selected on the basis of their confounding effect size, when introduced singly, on the β coefficient of the GlycA change-global cognition association (additionally adjusted for baseline measurement of GlycA and the time that elapsed between measurements). Although age and sex did not meet the criterion for inclusion, we retained adjustment for these variables. Social mobility, religiosity, anxiety, systolic and diastolic blood pressure, alcohol intake, fasting plasma glucose, insulin resistance (HOMA1-IR), homocysteine, total cholesterol, HDL-C, non HDL-C, LDL-C and triglycerides showed no material confounding effect and were not included. In a further step, analyses were additionally adjusted for change in smoking status and change in BMI during the 13 yr follow-up. To avoid loss of observations in the multivariable analyses, missing values were replaced with non-missing median (adult SES, n = 5; leisure-time vigorous activity, n = 1) or mean (depression, n = 4; change in BMI, n = 3) values.

Statistical analyses were carried out using SPSS v21.0 (IBM Corp., Armonk, NY).

A data file of the study variables can be found in the S1 Table.

## Results

Characteristics of the study sample are presented in Table 1. Participants were aged 28–32 at baseline (mean 30.1y) and 41–46 at follow-up (mean 43.1y) with a range of 12 to 16 years of follow-up (mean 13.0±0.7y); 32% were women, and 54% were high school or university graduates.

**Table 1:** Characteristics of the study sample: the Jerusalem LRC longitudinal study, 1976–2011.

Characteristics	Total
n	507
Socio-demographic variables	
Age at baseline (y) (range)	30.1±0.8 ^a^ (28.1–32.1)
Age at follow-up (y) (range)	43.1±0.9 (41.2–46.6)
Female (%)	32.3
Country of birth (%)	
Israel	22.5
Europe	22.7
Asia	29.4
N. Africa	25.4
Religiosity (%)	
Ultra-orthodox	7.9
Religious	19.1
Traditional	31.8
Secular	41.2
Education, highest level (%)	
University graduate	32.3
High school graduate	21.7
High school not graduated (9–12 yrs)	40.0
Elementary school (≤ 8 yrs)	5.9
Adult SES (ICBS ranking) ^b^	2.7 ± 1.2
Adult SES (MacArthur Scale) ^c^	7.2 ± 1.5
Childhood SES (ICBS ranking) ^b^	3.8 ± 1.6
Childhood SES (Vered Kraus Scale) ^c^	44.4 ± 30.1
Social mobility ^d^	1.1 ± 1.6
Inflammatory markers at baseline	
C-reactive protein (nmol/L)	21.2 ± 31.0
White blood cell count	6850 ± 1700
Fibrinogen (mg/dL)	233.2 ± 54.9
GlycA (μmol/L)	265 ± 52
GlycA change (μmol/L) ^e^	97± 54
Anthropometric and blood pressure at baseline	
BMI (kg/m^2^)	24.7 ± 3.6
BMI (kg/m^2^) at follow-up	27.1 ± 4.4
Systolic blood pressure (mmHg)	112 ± 10
Diastolic blood pressure (mmHg)	68 ± 9
Psychosocial variables	
Depressive symptoms score (HADS 0–21) ^f^	3.6 ± 2.9
Anxiety symptoms score (HADS 0–21) ^f^	5.6 ± 3.7
Lifestyle variables at baseline	
Leisure-time vigorous activity (%) ^g^	21.7
Alcohol intake of ≥once/ week (%)	39.4
Low intake (units/week) ^h^	1.4 ± 0.5
High intake (units/week) ^h^	5.7 ± 2.8
Pack-years (whole sample)	4.5 ± 6.5
Among ever smoked	9.5 ± 6.5
% current smokers	37.7
Pack-years at follow-up (whole sample)	8.3 ± 11.8
Among ever smoked	17.4 + 11.6
% current smokers	31.4
Biochemistry at baseline	
Plasma Lipids (mmol/L)	
Total cholesterol	4.4 ± 0.8
HDL-cholesterol	1.0 ± 0.3
Non-HDL-cholesterol	3.4 ± 0.9
LDL-cholesterol ^i^	2.7 ± 0.7
Triglycerides	1.4 ± 0.9
Fasting plasma glucose (mmol/L)	5.1 ± 0.5
HOMA-IR (mmol/L) ^j^	3.9 ± 1.8
Homocysteine (μmol/L)	12.2 ± 8.5

LRC, Lipid Research Clinic; BMI, body mass index

^a^ Mean ± SD (all such values).

^b^ An higher value infers a lower SES. Scale range from 1–6.

^c^ An higher value infers a higher SES. Vered Kraus scores range from 2.60–98.96; MacArthur Scale range from 1–10.

^d^ Computed by subtracting ICBS-based SES in adulthood from SES in childhood (both with a range from 1 (upper) to 6 (lower)). Range of social mobility score was from -5 (maximal downward drifting) to +5 (maximal upward mobility). No change/upward mobility corresponds to scores ≥0, whereas downward drifting corresponds to scores <0.

^e^ Computed by subtracting baseline GlycA at ages 28–32 from GlycA measured at ages 41–46.

^f^ 7-items each scored 0-3.Scale range from 0–21. Cronbach's alphas were adequate at .71 and .785 for the depression and the anxiety subscale, respectively.

^g^ Exercise for at least 20 minutes causing heavy breathing and sweating.

^h^ Low/ high intake, according to median split of alcohol intake among consumers of ≥once/ week.

^i^ Computed by the Friedewald method [38].; not computed for 7 males at age 30 and 11 participants (10 males and 1 females) at age 43 with triglycerides > 400 mg/dL.

^j^ Calculated as the product of fasting serum glucose (mmol/L) x fasting serum insulin (mlU/L) divided by 22.5.

Missing data: adult SES (ICBS ranking) (n = 5), adult SES (MacArthur Scale) (n = 11), early SEP (Vered Kraus Scale) (n = 4), social mobility (n = 5), depressive symptoms score (n = 4), anxiety symptoms score (n = 4), leisure-time vigorous activity (n = 1), BMI at follow-up (n = 1), LDL-cholesterol (n = 7), HOMA-IR (n = 1), homocysteine (n = 16), C-reactive protein (n = 14), WBC (n = 12), fibrinogen (n = 43), baseline GlycA (n = 21), GlycA change (n = 23).

Mean BMI increased from 24.7 ± 3.6 to 27.1 ± 4.4 during follow-up, while the smoking prevalence declined from 38% to 31%. Alcohol intake was low as was leisure-time vigorous activity. Mean HDL-cholesterol was low (as has been reported [27]).

Levels of GlycA, CRP, WBC and fibrinogen at baseline and GlycA change over the 13-year follow-up were available for 486, 493, 495, 464 and 484 of the 507 participants assessed subsequently for global cognitive function, respectively. Median values for GlycA, CRP, WBC and fibrinogen at baseline were 264 μmol/L (interquartile range [IQR]: 226 to 301μmol/L), 10.8 nmol/L (IQR: 5.0 to 25.5 nmol/L) (CRP conversion factor to mg/L units, divide by 9.524), 6,600 (IQR: 5600 to 7700) and 229 mg/dL (IQR: 194 to 265 mg/dL). GlycA correlated positively with CRP (Spearman rho = 0.55, p < .001) and less strongly with WBC and fibrinogen (rho = 0.33 and 0.32, respectively, p < .001, for both). Mean GlycA increased from 265 μmol/L at baseline to 361 μmol/L at follow-up. A GlycA increase over the mean 13 year follow-up period was evident in 473 of the 484 participants measured (98%), consistent with reports of a CRP increase with age [41, 42].

In unadjusted linear regression models of the baseline inflammatory markers, fibrinogen was inversely associated with global cognition and the specific domain of executive function, GlycA showed similar but weaker associations, whereas CRP and WBC were not associated with global cognition. Unadjusted increase in GlycA over 13 years was associated with lower global cognition, an association that was mostly evident in the upper quintile of GlycA change (p = 0.010 for trend in quintiles, and p = 0.003 for quintile 5 vs. the lower 4 quintiles grouped) (Table 2, Model 1). Multiple regression models were adjusted for age, sex, educational level, origin, childhood SES (ICBS ranking) and adult SES (ICBS ranking), baseline leisure-time vigorous activity, smoking status, BMI and depression (Table 2, Model 2). The association of global cognition with GlycA change, but not with the baseline inflammatory markers, persisted for the highest quintile of change (p for trend = .018 and p = 0.005 for the upper vs. the 4 lower quintiles grouped). The domains that contributed to this association were information processing speed (p = .012, upper vs. lower 4 quintiles) and memory (p = .007) (Table 2, Model 2).

**Table 2:** Linear regression of cognitive function in midlife (~50 y) on inflammatory markers at age ~30 y (baseline) and 13 y change in GlycA: the Jerusalem LRC longitudinal study.

Inflammatory marker ^f^	Global ^a^ (n = 507)			Attention ^a^ (n = 507)			Information processing speed (n = 477)			Executive (n = 506)			Visual spatial (n = 505)			Memory ^a^ (n = 499)		
	Model 1	Model 2	Model 3	Model 1	Model 2	Model 3	Model 1	Model 2	Model 3	Model 1	Model 2	Model 3	Model 1	Model 2	Model 3	Model 1	Model 2	Model 3
	β ^b^ (*p*)	β ^b^ (*p*)	β ^b^ (*p*)	β ^b^ (*p*)	β ^b^ (*p*)	β ^b^ (*p*)	β ^b^ (*p*)	β ^b^ (*p*)	β ^b^ (*p*)	β ^b^ (*p*)	β ^b^ (*p*)	β ^b^ (*p*)	β ^b^ (*p*)	β ^b^ (*p*)	β ^b^ (*p*)	β ^b^ (*p*)	β ^b^ (*p*)	β ^b^ (*p*)
1. CRP (nmol/L) ^a^ ^c^ at baseline	-.053	.012	.010	-.038	.008	.019	-.097*	-.061	-.044	-.080	-.033	-.019	-.036	.014	-.009	-.004	.044	.037
	(.243)	(.770)	(.809)	(.404)	(.865)	(.676)	(.036)	(.180)	(.342)	(.076)	(.473)	(.688)	(.426)	(.744)	(.833)	(.925)	(.343)	(.430)
2. WBC (cells/μL) ^c^ at baseline	-.007	.018	.014	.008	.032	.032	-.067	-.051	-.049	-.028	-.014	-.016	-.023	-.012	-.016	.004	.027	.021
	(.881)	(.659)	(.729)	(.862)	(.463)	(.466)	(.151)	(.255)	(.280)	(.528)	(.758)	(.720)	(.611)	(.784)	(.706)	(.922)	(.566)	(.650)
3. Fibrinogen (mg/dL) ^c^ at baseline	-.125**	-.036	-.031	-.053	.014	.022	-.048	.002	.011	-.138**	-.074	-.062	-.089	.007	.005	.091	-.065	.061
	(.007)	(.383)	(.467)	(.235)	(.760)	(.636)	(.317)	(.967)	(.821)	(.003)	(.107)	(.181)	(.055)	(.883)	(.918)	(.052)	(.169)	(.198)
4. GlycA (μmol/L) ^c^ at baseline	-.082	.006	.008	-.071	-.013	-.006	-.065	-.005	.008	-.104*	-.046	-.036	-.058	.006	-.003	.003	.082	.080
	(.072)	(.890)	(.857)	(.119)	(.768)	(.901)	(.163)	(.908)	(860)	(.022)	(.323)	(.448)	(.204)	(.886)	(.950)	(.948)	(.083)	(.095)
5. GlycA (μmol/L) change ^c^ ^d^	-.118**	-.086	-.076	-.079	-.059	-.067	-.109*	-.084	-.098	-.064	-.055	-.056	-.076	-.055	-.033	-.093	-.062	-.048
	(.023)	(.051)	(.112)	(.127)	(.218)	(.195)	(.042)	(.089)	(.063)	(.216)	(.263)	(.289)	(.142)	(.245)	(.511)	(.077)	(.214)	(.370)
6. GlycA (μmol/L) change (quintiles, 4df) ^d^																		
1 ≤ 49.88	0	0	0	0	0	0	0	0	0	0	0	0	0	0	0	0	0	0
2 49.89–83.21	-.050	-.050	-.048	-.078	-.083	-.083	-.017	-.030	-.030	-.049	-.058	-.057	-.101	-.108*	-.110*	-.007	-.003	-.002
	(.395)	(.323)	(.347)	(.183)	(.128)	(.132)	(.772)	(.587)	(.601)	(.401)	(.294)	(.313)	(.084)	(.045)	(.043)	(.901)	(.955)	(.967)
3 83.22–108.81	-.081	-.043	-.042	-.102	-.068	-.069	-.012	.015	.015	-.066	-.039	-.038	-.159**	-.134*	-.140*	.038	.066	.068
	(.171)	(.399)	(.418)	(.089)	(.217)	(.220)	(.842)	(.793)	(.798)	(.270)	(.489)	(.509)	(.008)	(.014)	(.011)	(.524)	(.249)	(.243)
4 108.82–139.19	-.034	-.032	-.027	-.032	-.034	-.040	.005	-.004	-.016	-.029	-.031	-.034	-.101	-.101	-.090	.018	.027	.034
	(.575)	(.543)	(.611)	(.602)	(.547)	(.499)	(.935)	(.943)	(.787)	(.637)	(.585)	(.568)	(.098)	(.069)	(.117)	(.765)	(.646)	(.579)
5 ≥ 139.20	-.186**	-.148**	-.143*	-.131	-.106	-.115	-.146	-.117	-.132*	-.117	-.111	-.116	-.145*	-.122*	-.111	-.134*	-.098	-.087
	(.003)	(.006)	(.015)	(.039)	(.072)	(.070)	(.025)	(.052)	(.040)	(.063)	(.065)	(.074)	(.022)	(.036)	(.074)	(.035)	(.109)	(.182)
*p* for trend ^d^	(.010)	(.018)	(.041)	(.181)	(.297)	(.277)	(.036)	(.077)	(.051)	(.141)	(.157)	(.167)	(.106)	(.180)	(.362)	(.038)	(.111)	(.205)
7. High GlycA (μmol/L) change (highest quintile, 1df) ^d^ ^e^	-.141**	-.114**	-.109*	-.073	-.056	-.060	-.140**	-.113*	-.124**	-.078	-.075	-.078	-.044	-.025	-.010	-.148**	-.123**	-.117*
	(.003)	(.005)	(.011)	(.122)	(.207)	(.194)	(.004)	(.012)	(.008)	(.099)	(.093)	(.098)	(.353)	(.561)	(.824)	(.002)	(.007)	(.014)

LRC, Lipid Research Clinic; Beta = standardized regression coefficient; CRP, C-reactive protein.

^a^ Box-Cox transformed z-scores (λ = - 0.5), C-reactive protein (λ = 0).

^b^ Standardized regression coefficient.

^c^ Per 1 SD increment.

^d^ Additionally adjusted for baseline measurement of GlycA and time elapsed between measurements.

^e^ Compared to the 4 lowest quintiles grouped.

Model 1: Unadjusted

Model 2: Adjusted for age, sex, educational level, origin, childhood SES (ICBS ranking) and adult SES (ICBS ranking), depression, and baseline leisure-time vigorous activity, smoking status and BMI measured at ages 28–32.

Model 3: Model 2 plus change in smoking status, change in BMI, and duration between measurements. Reference level of dummy variables: sex, females; educational level, elementary (<9 yrs.); origin, Israel; leisure-time vigorous activity, none; smoking status, never; change in smoking status, no change. Age, childhood SES, adult SES, depression, BMI, change in BMI; all introduced as continuous/ interval variables.

^f^ Missing data: CRP (n = 14), WBC (n = 12), fibrinogen (n = 43), GlycA at baseline (n = 21), GlycA change (n = 23), adult SES (n = 5), depression (n = 4), leisure-time vigorous activity (n = 1), change in BMI (n = 1). Missing values of adult SES, depression, leisure-time vigorous activity and change in BMI were replaced with non-missing median or mean values.

P-values in parentheses.

* p < .05.

** p < .01

Weight gain and cessation of smoking over the 13-year follow-up were associated with change in GlycA (not shown), indicating enhanced and reduced inflammatory responses, respectively, further supporting the role of GlycA as an inflammatory marker. Adjustment for change in smoking status and change in BMI during the follow-up had little effect on the magnitude of the associations (Table 2, Model 3). Additional adjustment for baseline triglyceride concentration and change in triglycerides did not affect the associations (not shown). Substituting mother's country of origin for father's country of origin did not materially alter the association of GlycA change with global cognition (not shown).

Using multivariable logistic models to predict poorer cognitive function (defined as the bottom fifth of the cognitive distribution), high GlycA change was associated with poorer global cognitive scores compared with the lowest quintile of GlycA change ((Table 3, model 2 adjusted OR = 4.44, 95%CI, 1.71–11.52, p = 0.002, and p for trend = 0.024, and model 3 OR = 4.81, 95%CI, 1.71–13.54, p = .003, and p for trend = 0.031). The major contribution to poorer global cognition was from the visual spatial domain (p trend = 0.014 and 0.067 for models 2 and 3 (Table 3), respectively. The comparison of the top quintile of GlycA change vs. the bottom 4 grouped yielded ORs of 2.52; 95%CI, 1.32–4.84, p = .005 for model 2 and 2.68; 95%CI, 1.34–5.39, p = .006 for model 3. The memory component contributed in model 2 (OR, 1.94; 95%CI, 1.03–3.65, p = .039), but less so in model 3. In summary, an enhanced inflammatory response between the ages of 30 and 43y was associated with poorer cognition at age 48–52.

**Table 3:** Logistic regression of low ranked cognitive function (lowest quintile) in midlife (~50 y) on inflammatory markers at age ~30 y (baseline) or ~43 y (follow-up) and 13 y change in GlycA: the Jerusalem LRC longitudinal study.

Inflammatory marker ^d^	Global (n = 507)			Attention (n = 507)			Information processing speed (n = 477)			Executive (n = 506)			Visual spatial (n = 505)			Memory (n = 499)		
	Model 1	Model 2	Model 3	Model 1	Model 2	Model 3	Model 1	Model 2	Model 3	Model 1	Model 2	Model 3	Model 1	Model 2	Model 3	Model 1	Model 2	Model 3
	OR[95% CI](*p*)	OR[95% CI](*p*)	OR[95% CI](*p*)	OR[95% CI](*p*)	OR[95% CI](*p*)	OR[95% CI](*p*)	OR[95% CI](*p*)	OR[95% CI](*p*)	OR[95% CI](*p*)	OR[95% CI](*p*)	OR[95% CI](*p*)	OR[95% CI](*p*)	OR[95% CI](*p*)	OR[95% CI](*p*)	OR[95% CI](*p*)	OR[95% CI](*p*)	OR[95% CI](*p*)	OR[95% CI](*p*)
1.CRP (nmol/L) ^a^	1.16	1.06	1.06	1.14	1.14	1.12	1.23*	1.21	1.18	1.24*	1.11	1.09	1.14	1.10	1.12	.99	.91	.91
	[.96–1.41]	[.83–1.34]	[.083–1.36]	[.94–1.39]	[.91–1.43]	[.089–1.41]	[1.01–1.50]	[.94–1.54]	[.91–1.52]	[1.02–1.51]	[.89–1.40]	[.86–1.38]	[.94–1.39]	[.87–1.40]	[.089–1.41]	[.81–1.20]	[.72–1.15]	[.072–1.15]
	(.131)	(.638)	(.640)	(.173)	(.258)	(.346)	(.040)	(.137)	(.206)	(.028)	(.356)	(.475)	(.197)	(.258)	(.437)	(.885)	(.427)	(.424)
2.WBC (10^3^/μL) at baseline	1.07	1.04	1.04	1.008	.982	.982	1.13	1.13	1.14	1.09	1.09	1.10	1.03	1.03	1.03	.96	.89	.90
	[.94–1.21]	[.89–1.23]	[.89–1.23]	[.89–1.15]	[.85–1.14]	[.84–1.14]	[.99–1.28]	[.97–1.32]	[.98–1.34]	[.97–1.24]	[.95–1.27]	[.95–1.28]	[.90–1.18]	[.88–1.21]	[.88–1.22]	[.84–1.10]	[.76–1.05]	[.77–1.06]
	(.290)	(.618)	(.611)	(.902)	(.812)	(.817)	(.058)	(.111)	(.093)	(.162)	(.224)	(.194)	(.663)	(.708)	(.694)	(.548)	(.173)	(.207)
3.Fibrinogen (mg/dL) at baseline	1.005*	1.004	1.004	1.002	1.000	1.000	1.001	1.000	.999	1.006**	1.005*	1.005	1.004	1.001	1.001	1.004*	1.004	1.004
	[1.001–1.009]	[.999–1.009]	[.999–1.009]	[.998–1.006]	[.996–1.005]	[.995–1.005]	[.997–1.005]	[.995–1.006]	[.994–1.005]	[1.002–1.011]	[1.000–1.009]	[1.000–1.009]	[1.000–1.008]	[.996–1.007]	[.996–1.007]	[1.000–1.008]	[.999–1.009]	[.999–1.009]
	(.023)	(.138)	(.141)	(.408)	(.922)	(.956)	(.633)	(.916)	(.846)	(.002)	(.042)	(.061)	(.061)	(.583)	(.639)	(.037)	(.118)	(.143)
4.GlycA (μmol/L) at baseline	1.003	0.999	0.998	1.003	1.002	1.002	1.003	1.000	.999	1.007**	1.005*	1.005	1.004	1.002	1.002	1.003	1.000	1.000
	[.998–1.007]	[.993–1.004]	[.993–1.004]	[.999–1.008]	[.997–1.007]	[.996–1.007]	[.999–1.007]	[.995–1.005]	[.994–1.005]	[1.002–1.012]	[1.000–1.011]	[1.000–1.010]	[.999–1.008]	[.996–1.007]	[.996–1.007]	[.998–1.007]	[.995–1.006]	[.995–1.006]
	(.206)	(.622)	(.536)	(.120)	(.379)	(.542)	(.180)	(.951)	(.831)	(.002)	(.047)	(.062)	(.089)	(.533)	(.503)	(.244)	(.862)	(.911)
5.GlycA (μmol/L) change ^b^	1.006*	1.007*	1.007*	1.003	1.003	1.003	1.002	1.002	1.002	1.002	1.002	1.004	1.005*	1.006*	1.004	1.004	1.004	1.004
	[1.001–1.011]	[1.000–1.012]	[1.000–1.013]	[.999–1.008]	[.998–1.008]	[.997–1.008]	[.997–1.007]	[.996–1.007]	[.996–1.008]	[.997–1.007]	[.997–1.007]	[.996–1.010]	[1.000–1.010]	[1.000–1.011]	[.998–1.010]	[.999–1.009]	[.999–1.009]	[.999–1.009]
	(.014)	(.022)	(.035)	.143	.197	.313	.382	.545	.501	.348	.397	.175	.041	.040	.194	.099	.144	.362
6.GlycA (μmol/L) change (quintiles, 4df) ^b^																		
1 ≤ 49.88	1	1	1	1	1	1	1	1	1	1	1	1	1	1	1	1	1	1
2 49.89–83.21	2.19*	2.75*	2.72*	1.97	2.07	1.96	.79	.92	.90	1.89	2.28	2.53	2.94*	2.94*	2.81*	1.48	1.62	1.54
	[1.01–4.73]	[1.13–6.71]	[1.10–6.71]	[.92–4.19]	[.92–4.65]	[.86–4.5]	[.38–1.65]	[.41-.07]	[.39–2.06]	[.92–3.89]	[1.03-.03]	[1.12-.71]	[1.01–5.21]	[1.17–7.40]	[1.11–7.14]	[.72–3.06]	[.72–3.65]	[.68–3.50]
	(.046)	(.026)	(.030)	(.079)	(.079)	(.109)	(.535)	(.838)	(.799)	.083	.042	(.025)	(.047)	(.022)	(.030)	(.288)	(.242)	(.302)
3 83.22–108.81	1.95	1.62	1.60	2.24*	1.96	1.82	.85	.68	.67	1.45	1.34	1.48	2.49*	2.60*	2.50	1.06	.92	.86
	[0.88–4.32]	[0.64–4.11]	[0.62–4.15]	[1.04–4.79]	[.86–4.46]	[.79–4.21]	[.41–1.77]	[.30–1.57]	[.28–1.58]	[.68–3.11]	[.58–3.08]	[.63–3.48]	[1.08–5.72]	[1.02–6.63]	[.97–6.49]	[.49–2.31]	[.39–2.19]	[.36–2.09]
	(.101)	(.313)	(.332)	(.039)	(.109)	.162	(.667)	(.372)	(.362)	.341	.495	(.371)	(.032)	(.046)	(.059)	(.877)	(.847)	(.742)
4 108.82–139.19	1.44	1.61	1.60	1.61	1.73	1.65	.75	.76	.76	1.46	1.71	2.09	2.74**	3.54**	3.10*	1.12	1.09	.97
	[0.61–3.38]	[0.61–4.26]	[0.58–4.41]	[.71–3.63]	[.73–4.12]	[.67–4.04]	[.34–1.65]	[.32–1.81]	[.31–1.88]	[.66–3.22]	[.72–4.03]	[.85–5.15]	[1.17–6.42]	[1.37–9.20]	[1.16–8.31]	[.50–2.31]	[.45–2.63]	[.39–2.41]
	(.404)	(.339)	(.360)	(.252)	(.214)	(.278)	(.481)	(.542)	(.550)	.345	.224	(.110)	(.020)	(.009)	(.025)	(.849)	(.849)	(.941)
5 ≥ 139.20	3.86**	4.44**	4.81**	2.47*	2.36	2.20	1.28	1.20	1.26	1.82	1.90	2.47	3.51**	4.34**	3.73*	2.24	2.24	1.87
	[1.72–8.65]	[1.71–11.52]	[1.71–13.54]	[1.10–5.56]	[.99–5.62]	[.87–5.61]	[.59–2.76]	[.51–2.84	[.49–3.21]	[.81–4.08]	[.78–4.58]	[.95–6.46]	[1.47–8.35]	[1.64–11.51]	[1.31–10.65]	[.934–5.39]	[.934–5.39]	[.731–4.80]
	(.001)	(.002)	(.003)	(.029)	.053	(.097)	(.531)	(.676)	(.633)	.146	.155	(.064)	(.005)	(.003)	(.014)	(.071)	(.071)	(.191)
*p* for trend ^b^	(.012)	(.024)	(.031)	(.155)	(.219)	(.329)	(.444)	(.633)	(.562)	(.442)	(.494)	(.276)	(.016)	(.014)	(.067)	(.136)	(.179)	(.417)
7.High GlycA (μmol/L) change (highest quintile, 1df) ^b^ ^c^	2.32**	2.52**	2.68**	1.42	1.36	1.29	1.54	1.49	1.61	1.24	1.20	1.33	1.60	1.67	1.43	1.83*	194*	1.75
	[1.34–4.01]	[1.32–4.84]	[1.34–5.39]	[.81–2.51]	[.74–2.49]	[.68–2.46]	[.85–2.77]	[.78–2.84]	[.81–3.18]	[.68–2.27]	[.63–2.29]	[.67–2.63]	[.89–2.86]	[.87–3.20]	[.71–2.87]	[1.04–3.22]	[1.03–3.65]	[.90–3.38]
	(.003)	(.005)	(.006)	(.224)	(.327)	(.432)	(.153)	(.234)	(.173)	(.485)	(.588)	(.415)	(.117)	(.121)	(.314)	(.037)	(.039)	(.097)

LRC, Lipid Research Clinic; CRP, C-reactive protein.

^a^ Box-Cox transformed CRP (λ = 0).

^b^ Additionally adjusted for baseline measurement of GlycA and time elapsed between measurements.

^c^ Compared to the 4 lowest quintiles grouped.

Model 1: Unadjusted

Model 2: Adjusted for age, sex, educational level, origin, childhood SES (ICBS ranking) and adult SES (ICBS ranking), depression, and baseline leisure-time vigorous activity, smoking status and BMI measured at ages 28–32.

Model 3: Model 2 plus change in smoking status, change in BMI, and duration between measurements. Reference level of dummy variables: sex, females; educational level, elementary (<9 yrs.); origin, Israel; leisure-time vigorous activity, none; smoking status, never; change in smoking status, no change. Age, childhood SES, adult SES, depression, BMI, change in BMI; all introduced as continuous/ interval variables.

^d^ Missing data: CRP (n = 14), white blood cell count (n = 12), fibrinogen (n = 43), GlycA at baseline (n = 21), GlycA change (n = 23), adult SES (n = 5), depression (n = 4), leisure-time vigorous activity (n = 1), change in BMI (n = 1). Missing values of adult SES, depression, leisure-time vigorous activity and change in BMI were replaced with non-missing median or mean values.

P-values in parentheses.

* p < .05.

** p < .01

## Discussion

To the best of our knowledge this is the first study to evaluate associations of baseline levels and long-term change in inflammation status with cognition in healthy young adults. We examined baseline CRP, WBC, fibrinogen and GlycA levels at ages 28–32 and GlycA change over a 13-year interval in a sample of the Jerusalem LRC Study cohort. Our results indicate that an enhanced inflammatory response between the ages of 30 and 43 years was associated with poorer cognitive function at age 48–52, whereas baseline inflammation level at a young age was not predictive of midlife cognition. The associations were robust to adjustment for sex, socio-demographic characteristics, smoking, BMI, exercise, depression and plasma triglycerides, and persisted after further adjustment for change in smoking status, change in BMI and change in triglycerides during the 13y follow-up.

To date few studies have examined longitudinal change in inflammation in relation to cognition. Our results regarding GlycA change-cognition associations are consistent with those recently reported in a sample of over 1,600 adults aged 65 and older from the US Cardiovascular Health Study All Stars where increase in CRP or IL-6 over 9 years was associated with increased risk of cognitive impairment. Associations were observed with the Modified Mini-Mental Status Examination (3MSE) and the digit symbol substitution test (DSST), the former reflecting global cognitive function and the latter as a test of sustained attention, response speed and visuomotor coordination [14]. In the Jerusalem LRC study, however, attention, visual spatial and executive function did not appear to be the domains associated with long-term inflammation, but rather global cognitive function and its information processing speed and memory domains. In contrast, the Whitehall II study of over 5,000 British civil servants aged 45–69 at baseline measurement of CRP and IL-6, showed no association of change in the inflammatory markers over a 6-yr interval with any of the cognitive domain scores [17].

Other studies investigated inflammatory marker levels measured at a single time point. Some reported null associations generally consistent with our findings for baseline inflammation [6, 7], although most reported inverse associations with different and inconsistent sets of cognitive domains between studies [8,14,17,9,10,13, 16,19,11,18] and/ or a measure of global cognitive function [5,17,12,16]. All these studies assessed individuals who were middle-aged and older[5,8–11, 17] or were elderly [10,12–16, 18, 19] at the time of inflammatory marker measurement, mostly of CRP and/ or IL-6 biomarkers [17,14,9,19,15,16,11], making it difficult to compare our baseline findings to others.

Several potential mechanisms might explain an association between inflammation and cognitive function. It may be that inflammation is associated with cognition indirectly through vascular mechanisms. Inflammation is associated with atherosclerosis/cardiovascular disease [43], including cerebrovascular disease [44], which could contribute to cognitive impairment. It has also been suggested that inflammation affects cognition through its impact on cerebral small-vessel disease [45] as postulated by the association with silent brain infarction [46].

It is also possible that inflammation is associated with cognitive function independently of vascular-related conditions. Accumulating evidence indicates that inflammation plays a role in neurodegenerative diseases and impaired memory through increased Aβ accumulation as reported in *APP* mice[47], as well as in numerous healthy-state cognitive processes through direct effects on synaptic plasticity, neurogenesis, and neuromodulation that affect cognition [48]. Some evidence indicates that peripheral cytokines penetrate the blood-brain barrier directly via active transport mechanisms or indirectly via vagal nerve stimulation [49].

The strengths of this study lie in the longitudinal assessment of change in a marker of inflammation, the comprehensive objective computerized cognitive measures with millisecond precision [50], and the wide range of potential confounders evaluated, including detailed socio-demographic, psychosocial, anthropometric, health behavioral and laboratory variables. The young age at baseline inflammation measurement and the age of the participants at cognitive testing, which are younger than in most studies on cognitive aging [51], differ from previous research on this topic. Further strengths are the use of 4 markers of systemic inflammation, including the use of the novel NMR-determined inflammatory biomarker, GlycA, and accounting for SES indices both in childhood and adulthood as well as changes in SES, smoking status and BMI during the follow-up. A limitation is that the measures of cognition were done at one point in midlife. Consequently, no direct inference about the role of inflammation on cognitive decline can be drawn. However, the results of our study are unlikely to be affected by preclinical dementia common to previous reports of older individuals. Another limitation is the modest sample size (n = 484 for repeated measures of GlycA).

In conclusion, this study may be the first to address and identify in young adults an association of change in inflammation level over a long-term period with cognitive function. Additional longitudinal studies of young cohorts with measurement of GlycA and additional inflammatory markers are needed to confirm these findings. This study is consistent with a mechanism underlining the interplay between the immune system and cognitive decline and the recently proposed hypothesis that interventions through anti-inflammatory strategies [47, 52] may be therapeutically relevant to delay cognitive decline.

## Supporting Information

S1 File Description of the NeuroTrax computerized cognitive testing battery. (PDF)

S1 Table Supporting data file from the Jerusalem LRC. (XLSX)

## References

[1] Gorelick PB. Role of inflammation in cognitive impairment: results of observational epidemiological studies and clinical trials. Ann N Y Acad Sci. 2010;1207:155–62. 10.1111/j.1749-6632.2010.05726.x 20955439

[2] Tan ZS, Beiser AS, Vasan RS, Roubenoff R, Dinarello CA, Harris TB, et al Inflammatory markers and the risk of Alzheimer disease: the Framingham Study. Neurology. 2007;68:1902–8. 17536046 10.1212/01.wnl.0000263217.36439.da

[3] Koyama A, O'Brien J, Weuve J, Blacker D, Metti AL, Yaffe K. The role of peripheral inflammatory markers in dementia and Alzheimer's disease: a meta-analysis. J Gerontol A Biol Sci Med Sci. 2013;68:433–40. 10.1093/gerona/gls187 22982688 PMC3693673

[4] Engelhart MJ, Geerlings MI, Meijer J, Kiliaan A, Ruitenberg A, van Swieten JC, et al Inflammatory proteins in plasma and the risk of dementia: the rotterdam study. Arch Neurol. 2004;61:668–72. 15148142 10.1001/archneur.61.5.668

[5] Dik MG, Jonker C, Hack CE, Smit JH, Comijs HC, Eikelenboom P. Serum inflammatory proteins and cognitive decline in older persons. Neurology. 2005;64:1371–7. 15851726 10.1212/01.WNL.0000158281.08946.68

[6] Alley DE, Crimmins EM, Karlamangla A, Hu P, Seeman TE. Inflammation and rate of cognitive change in high-functioning older adults. J Gerontol A Biol Sci Med Sci. 2008;63:50–5. 18245760 10.1093/gerona/63.1.50 PMC2952346

[7] Weuve J, Ridker PM, Cook NR, Buring JE, Grodstein F. High-sensitivity C-reactive protein and cognitive function in older women. Epidemiology. 2006;17:183–9. 16477259 10.1097/01.ede.0000198183.60572.c9

[8] Teunissen CE, van Boxtel MP, Bosma H, Bosmans E, Delanghe J, De Bruijn C, et al Inflammation markers in relation to cognition in a healthy aging population. J Neuroimmunol. 2003;134:142–50. 12507782 10.1016/s0165-5728(02)00398-3

[9] Windham BG, Simpson BN, Lirette S, Bridges J, Bielak L, Peyser PA, et al Associations between inflammation and cognitive function in african americans and European americans. J Am Geriatr Soc. 2014;62:2303–10. 10.1111/jgs.13165 25516026 PMC4270090

[10] Kao TW, Chang YW, Chou CC, Hu J, Yu YH, Kuo HK. White blood cell count and psychomotor cognitive performance in the elderly. Eur J Clin Invest. 2011;41:513–20. 10.1111/j.1365-2362.2010.02438.x 21466549

[11] Gimeno D, Marmot MG, Singh-Manoux A. Inflammatory markers and cognitive function in middle-aged adults: the Whitehall II study. Psychoneuroendocrinology. 2008;33:1322–34. 10.1016/j.psyneuen.2008.07.006 18774232 PMC2613425

[12] Schram MT, Euser SM, de Craen AJ, Witteman JC, Frolich M, Hofman A, et al Systemic markers of inflammation and cognitive decline in old age. J Am Geriatr Soc. 2007;55:708–16. 17493190 10.1111/j.1532-5415.2007.01159.x

[13] Jefferson AL, Massaro JM, Beiser AS, Seshadri S, Larson MG, Wolf PA, et al Inflammatory markers and neuropsychological functioning: the Framingham Heart Study. Neuroepidemiology. 2011;37:21–30. 10.1159/000328864 21757961 PMC3142099

[14] Jenny NS, French B, Arnold AM, Strotmeyer ES, Cushman M, Chaves PH, et al Long-term assessment of inflammation and healthy aging in late life: the Cardiovascular Health Study All Stars. J Gerontol A Biol Sci Med Sci. 2012;67:970–6. 10.1093/gerona/glr261 22367431 PMC3436091

[15] Noble JM, Manly JJ, Schupf N, Tang MX, Mayeux R, Luchsinger JA. Association of C-reactive protein with cognitive impairment. Arch Neurol. 2010;67:87–92. 10.1001/archneurol.2009.308 20065134 PMC4426905

[16] Rafnsson SB, Deary IJ, Smith FB, Whiteman MC, Rumley A, Lowe GD, et al Cognitive decline and markers of inflammation and hemostasis: the Edinburgh Artery Study. J Am Geriatr Soc. 2007;55:700–7. 17493189 10.1111/j.1532-5415.2007.01158.x

[17] Singh-Manoux A, Dugravot A, Brunner E, Kumari M, Shipley M, Elbaz A, et al Interleukin-6 and C-reactive protein as predictors of cognitive decline in late midlife. Neurology. 2014;83:486–93. 10.1212/WNL.0000000000000665 24991031 PMC4141998

[18] Rafnsson S, Deary IJ, Whiteman MC, Rumley A, Lowe GD, Fowkes FG. Haemorheological predictors of cognitive decline: the Edinburgh Artery Study. Age Ageing. 2010;39:217–22. 10.1093/ageing/afp227 20097662

[19] Palta P, Xue QL, Deal JA, Fried LP, Walston JD, Carlson MC. Interleukin-6 and C-reactive protein levels and 9-year cognitive decline in community-dwelling older women: The Women's Health and Aging Study II. J Gerontol A Biol Sci Med Sci. 2014 [Epub ahead of print] 10.1093/gerona/glu132 25161214 PMC4481686

[20] Quinn TJ, Gallacher J, Deary IJ, Lowe GD, Fenton C, Stott DJ. Association between circulating hemostatic measures and dementia or cognitive impairment: systematic review and meta-analyzes. J Thromb Haemost. 2011;9:1475–82. 10.1111/j.1538-7836.2011.04403.x 21676170

[21] Otvos JD, Shalaurova I, Wolak-Dinsmore J, Connelly MA, Mackey RH, Stein JH, et al GlycA: a composite nuclear magnetic resonance biomarker of systemic inflammation. Clin Chem. 2015;61:714–23. 10.1373/clinchem.2014.232918 25779987

[22] de Heredia FP, Gomez-Martinez S, Marcos A. Obesity, inflammation and the immune system. Proc Nutr Soc. 2012;71:332–8. 10.1017/S0029665112000092 22429824

[23] Rom O, Avezov K, Aizenbud D, Reznick AZ. Cigarette smoking and inflammation revisited. Respir Physiol Neurobiol. 2013;187:5–10. 10.1016/j.resp.2013.01.013 23376061

[24] Dowd JB, Haan MN, Blythe L, Moore K, Aiello AE. Socioeconomic gradients in immune response to latent infection. Am J Epidemiol. 2008;167:112–20. 17873099 10.1093/aje/kwm247

[25] Slater PE, Friedlander Y, Baras M, Harlap S, Halfon ST, Kaufmann NA, et al The Jerusalem Lipid Research Clinic: sampling, response and selected methodological issues. Isr J Med Sci. 1982;18:1106–12. 7161042

[26] Kark JD, Sinnreich R, Leitersdorf E, Friedlander Y, Shpitzen S, Luc G. Taq1B CETP polymorphism, plasma CETP, lipoproteins, apolipoproteins and sex differences in a Jewish population sample characterized by low HDL-cholesterol. Atherosclerosis. 2000;151:509–18. 10924728 10.1016/s0021-9150(99)00408-6

[27] Kark JD, Goldberger N, Kimura M, Sinnreich R, Aviv A. Energy intake and leukocyte telomere length in young adults. Am J Clin Nutr. 2012;95:479–87. 10.3945/ajcn.111.024521 22237065 PMC3260074

[28] Dwolatzky T, Whitehead V, Doniger GM, Simon ES, Schweiger A, Jaffe D, et al Validity of a novel computerized cognitive battery for mild cognitive impairment. BMC Geriatr. 2003;3:4 14594456 10.1186/1471-2318-3-4 PMC270050

[29] Melton JL. Psychometric evaluation of the Mindstreams neuropsychological screening tool. NEDU Technical Report 06–10, Navy Experimental Diving Unit, Panama City, FL.2005.

[30] Schweiger A, Dwolatzky T, Jaffe D, Simon ES. Reliability of a novel computerized neuropsychological battery for mild cognitive impairment. Acta Neuropsychologica. 2003;1:407–13.

[31] Thaler A, Mirelman A, Gurevich T, Simon E, Orr-Urtreger A, Marder K, et al Lower cognitive performance in healthy G2019S LRRK2 mutation carriers. Neurology. 2012;79:1027–32. 22914834 10.1212/WNL.0b013e3182684646 PMC3430708

[32] Sasson E, Doniger GM, Pasternak O, Tarrasch R, Assaf Y. White matter correlates of cognitive domains in normal aging with diffusion tensor imaging. Front Neurosci. 2013;7:32 10.3389/fnins.2013.00032 23493587 PMC3595518

[33] Boussi-Gross R, Golan H, Volkov O, Bechor Y, Hoofien D, Schnaider Beeri M, et al Improvement of Memory Impairments in Poststroke Patients by Hyperbaric Oxygen Therapy. Neuropsychology. 2014 [Epub ahead of print] 10.1037/neu0000149 25384125

[34] Hartman SJ, Marinac CR, Natarajan L, Patterson RE. Lifestyle factors associated with cognitive functioning in breast cancer survivors. Psychooncology. 2014 [Epub ahead of print] 10.1002/pon.3626 25073541 PMC4310811

[35] Zur D, Naftaliev E, Kesler A. Evidence of Multidomain Mild Cognitive Impairment in Idiopathic Intracranial Hypertension. J Neuroophthalmol. 2015;35:26–30. 10.1097/WNO.0000000000000199 25383589

[36] Kraus V, Schild EO, Hodge RW. Occupational prestige in the collective conscience. Soc Forces. 1978;56:900–18.

[37] Burazeri G, Goda A, Sulo G, Stefa J, Kark JD. Financial loss in pyramid savings schemes, downward social mobility and acute coronary syndrome in transitional Albania. J Epidemiol Community Health. 2008;62:620–6. 10.1136/jech.2007.066001 18559445

[38] Friedewald WT, Levi RI, Fredrickson DS. Estimation of the concentration of low-density lipoprotein cholesterol in plasma, without use of the preparative ultracentrifuge. Clin Chem. 1972;18:499–502. 4337382

[39] Zigmond AS, Snaith RP. The hospital anxiety and depression scale. Acta Psychiatr Scand. 1983;67:361–70. 6880820 10.1111/j.1600-0447.1983.tb09716.x

[40] Sakia RM. The Box-Cox transformation technique: a review. The Statistician. 1992;41:169–78.

[41] Rumley A, Emberson JR, Wannamethee SG, Lennon L, Whincup PH, Lowe GD. Effects of older age on fibrin D-dimer, C-reactive protein, and other hemostatic and inflammatory variables in men aged 60–79 years. J Thromb Haemost. 2006;4:982–7. 16689748 10.1111/j.1538-7836.2006.01889.x

[42] Hutchinson WL, Koenig W, Frohlich M, Sund M, Lowe GD, Pepys MB. Immunoradiometric assay of circulating C-reactive protein: age-related values in the adult general population. Clin Chem. 2000;46:934–8. 10894836

[43] Pearson TA, Mensah GA, Alexander RW, Anderson JL, Cannon RO 3rd, Criqui M, et al Markers of inflammation and cardiovascular disease: application to clinical and public health practice: A statement for healthcare professionals from the Centers for Disease Control and Prevention and the American Heart Association. Circulation. 2003;107:499–511. 12551878 10.1161/01.cir.0000052939.59093.45

[44] Tarkowski E, Rosengren L, Blomstrand C, Wikkelso C, Jensen C, Ekholm S, et al Intrathecal release of pro- and anti-inflammatory cytokines during stroke. Clin Exp Immunol. 1997;110:492–9. 9409656 10.1046/j.1365-2249.1997.4621483.x PMC1904815

[45] Marchesi VT. Alzheimer's dementia begins as a disease of small blood vessels, damaged by oxidative-induced inflammation and dysregulated amyloid metabolism: implications for early detection and therapy. Faseb j. 2011;25:5–13. 10.1096/fj.11-0102ufm 21205781

[46] Hoshi T, Kitagawa K, Yamagami H, Furukado S, Hougaku H, Hori M. Relations of serum high-sensitivity C-reactive protein and interleukin-6 levels with silent brain infarction. Stroke. 2005;36:768–72. 15746456 10.1161/01.STR.0000158915.28329.51

[47] Chakrabarty P, Li A, Ceballos-Diaz C, Eddy JA, Funk CC, Moore B, et al IL-10 alters immunoproteostasis in APP mice, increasing plaque burden and worsening cognitive behavior. Neuron. 2015;85:519–33. 10.1016/j.neuron.2014.11.020 25619653 PMC4320003

[48] McAfoose J, Baune BT. Evidence for a cytokine model of cognitive function. Neurosci Biobehav Rev. 2009;33:355–66. 10.1016/j.neubiorev.2008.10.005 18996146

[49] Wilson CJ, Finch CE, Cohen HJ. Cytokines and cognition—the case for a head-to-toe inflammatory paradigm. J Am Geriatr Soc. 2002;50:2041–56. 12473019 10.1046/j.1532-5415.2002.50619.x

[50] Parsey CM, Schmitter-Edgecombe M. Applications of technology in neuropsychological assessment. Clin Neuropsychol. 2013;27:1328–61. 10.1080/13854046.2013.834971 24041037 PMC3869883

[51] Singh-Manoux A, Kivimaki M, Glymour MM, Elbaz A, Berr C, Ebmeier KP, et al Timing of onset of cognitive decline: results from Whitehall II prospective cohort study. BMJ. 2012;344:d7622 10.1136/bmj.d7622 22223828 PMC3281313

[52] Guillot-Sestier MV, Doty KR, Gate D, Rodriguez J Jr, Leung BP, Rezai-Zadeh K, et al Il10 deficiency rebalances innate immunity to mitigate Alzheimer-like pathology. Neuron. 2015;85:534–48. 10.1016/j.neuron.2014.12.068 25619654 PMC4352138

