# Supplementary material for: Increase in the Inflammatory Marker GlycA over 13 Years in Young Adults Is Associated with Poorer Cognitive Function in Midlife
Source: PLoS One. 2015 Sep 25;10(9):e0138036. doi: 10.1371/journal.pone.0138036 (PMC4583455; doi:10.1371/journal.pone.0138036)
Supplement: S1 File — (PDF) [file pone.0138036.s001.pdf]

Supporting Information

S1 File. Description of the NeuroTrax computerized cognitive testing battery.

Following is a brief description of the cognitive tests and the outcome parameters comprising the cognitive domains included in the current study. *(a) The executive function domain* included composite scores from the Go-NoGo response inhibition test, Stroop interference test at phase III, and Catch Game. The Go-NoGo test is a continuous performance test of simple response time and response inhibition. In the Stroop interference test, the conflicting information provided by the meaning of a word and the color of its letters leads to a decrement in performance relative to the test phases where there is no conflict. The test includes three phases-phase I (no interference: letter color), phase II (no interference: word meaning), and phase III (interference between letter color and word meaning). The Catch Game is a novel motor test requiring hand-eye coordination, scanning and rapid responses. Participants were instructed to 'catch' a rectangular white object falling vertically from the top of the screen by positioning a green rectangular 'paddle' moving horizontally in the path of the falling object. The executive function domain is an average of composite scores from the Go-NoGo response inhibition test and Stroop interference test at phase III, and mean accuracy for the Catch Game; *(b) The memory domain* included immediate and delayed recognition memory tests for the orientation of simple geometric patterns and symbols. Participants were presented with eight black-and-white drawn pictures of simple common objects and were instructed to remember their orientation. Up to four consecutive repetitions tests were administered, followed by a delayed recognition test following one intervening NeuroTrax test. The memory domain is an average of the accuracy on the four immediate repetitions and the delayed phase of the test; (*c) The information processing speed domain* comprised measures of information processing at increasing levels of complexity. The test was comprised of three blocks of information processing load: single digits (block 1), two-digit arithmetic problems (e.g., 5 – 1, block 2), and three-digit arithmetic problems (e.g., 3 + 2 – 1, block 3). For each of these three blocks, stimuli were presented at three different rates (speed levels), incrementally increasing. Participants were instructed to respond by pressing the left mouse button if the digit or result is less than or equal to 4 and the right mouse button if it is greater than 4. The information processing speed domain is a weighted average of the composite scores in block 1 at speed levels 1(weighted x0.10) and 3 (x0.2) and in block 2 at levels 1(x0.30) and 2 (x0.40); *(d) The attention domain* was computed as the average of: the mean response time of the Go-NoGo test, the response time standard deviation of the Go-NoGo, the mean response time of the Stroop interference (phase II) and staged information processing speed (block 1, level 2) test, as well as the accuracy score of the latter (block 2, level 3); *(e) The visual spatial processing domain* assesses abstract spatial ability. Participants

were presented with familiar daily scenes containing a red pillar (rectangle) and instructed to imagine standing at the location of the red pillar. Four views of the scene were presented and participants were required to indicate which of the four views corresponds to the view of the scene from the location of the pillar. The visual spatial domain is an accuracy score; *(f) The global cognitive* score was computed as the average of the domain scores, thus reflecting overall cognitive function.
